# Targeted irradiation in an autochthonous mouse model of pancreatic cancer

**DOI:** 10.1242/dmm.050463

**Published:** 2024-03-14

**Authors:** Mathias Tesson, Katrina Stevenson, Saadia A. Karim, Colin Nixon, Anthony J. Chalmers, Owen J. Sansom, Eric O'Neill, Keaton Jones, Jennifer P. Morton

**Affiliations:** ^1^ CRUK Scotland Institute, Glasgow, G61 1BD, UK; ^2^ School of Cancer Sciences, University of Glasgow, Glasgow, G61 1QH, UK; ^3^ Department of Oncology, University of Oxford, Oxford, OX3 7DQ, UK

**Keywords:** Pancreatic cancer, Radiotherapy, Mouse models, Preclinical trials

## Abstract

The value of radiotherapy in the treatment of pancreatic cancer has been the subject of much debate but limited preclinical research. We hypothesise that the poor translation of radiation research into clinical trials of radiotherapy in pancreatic cancer is due, in part, to inadequate preclinical study models. Here, we developed and refined methods for targeted irradiation in autochthonous mouse models of pancreatic cancer, using a small animal radiotherapy research platform. We tested and optimised strategies for administration of contrast agents, iohexol and the liver imaging agent Fenestra LC, to enable the use of computed tomography imaging in tumour localisation. We demonstrate accurate tumour targeting, negligible off-target effects and therapeutic efficacy, depending on dose, number of fractions and tumour size, and provide a proof of concept that precise radiation can be delivered effectively to mouse pancreatic tumours with a clinically relevant microenvironment. This advance will allow investigation of the radiation response in murine pancreatic cancer, discovery of mechanisms and biomarkers of radiosensitivity or resistance, and development of radiosensitising strategies to inform clinical trials for precision radiotherapy in this disease.

## Teaser

**Summary:** Preclinical modelling is needed to better understand the effects of radiotherapy in pancreatic cancer. We optimised methods for and tested the efficacy of targeted irradiation in a clinically relevant autochthonous mouse model.

## INTRODUCTION

Pancreatic ductal adenocarcinoma (PDAC) is a highly aggressive malignancy with few treatment options. Despite multimodal treatment, the 5-year survival rate remains less than 10%, making pancreatic cancer the most lethal of all common cancers. Indeed, pancreatic cancer is predicted to become the second leading cause of cancer death within a decade, highlighting the urgent need to improve treatment options (Rahib et al., 2021). Rapid progression of the disease, coupled with late diagnosis, results in a median survival of less than 6 months for patients. Surgery remains the only potentially curative treatment, and in resectable or borderline resectable disease, neoadjuvant therapy may increase the likelihood of a margin-negative (R0) resection. In selected centres, particularly in North America, neoadjuvant chemotherapy may be followed by radiotherapy (RT). RT is most often delivered to patients with pancreatic cancer as conventional targeted RT, which consists of daily fractions of approximately 2-3 Gy delivered 5 days a week with a total dose ranging from 30 to 60 Gy. Computed tomography (CT) imaging of the abdomen is used to define the target tumour volume and adjacent healthy tissues to be spared. However, defining target volumes can be challenging owing to changes in tumour size and shape, as well as dynamic variations caused by normal physiology, for example, breathing, peristalsis and bladder size (Falco et al., 2023). Recent technical advances in CT technology and planning techniques that can account for tumour mobility have enabled more accurate delivery of radiation and the use of hypofractionation (a smaller number of fractions with increased dose per fraction) to increase tumour control while avoiding toxicity. Indeed, single doses of up to 25 Gy have been reported (Reyngold et al., 2019).

There is some evidence that neoadjuvant RT confers better locoregional control than chemotherapy alone, better pathological response and decreased lymph node involvement. However, this has not always translated to improved overall survival, and the utility of RT in the clinical management of pancreatic cancer is the subject of considerable debate, with several trials reporting conflicting results (Franko et al., 2017; Janssen et al., 2022; Lutfi et al., 2017; Nagakawa et al., 2019). For example, the LAP07 clinical trial found that RT delayed local tumour progression without improving overall survival when combined with chemotherapy (Hammel et al., 2016). In contrast, although the comparison of neoadjuvant and adjuvant therapy precludes evaluation of the impact of RT, the PREOPANC phase III trial reported that neoadjuvant gemcitabine combined with RT improved overall survival in cases of resectable or borderline resectable disease, with a 5-year survival by intention to treat of 20.5%, compared to 6.5% for upfront surgery with adjuvant gemcitabine. The R0 resection rate was also increased to 71% in patients receiving neoadjuvant chemoradiotherapy (CRT) compared to 40% in patients undergoing upfront surgery (Versteijne et al., 2020, 2022).

Failure to improve overall survival using RT in many cases, despite local control, has been ascribed to undetected metastasis at diagnosis. Therefore, the current view is to offer RT to patients unlikely to benefit from surgery or patients with locally advanced disease with a high risk of locoregional progression (Maxwell and Katz, 2021). However, the lack of conclusive clinical trial data directly assessing the role of RT in the perioperative management of pancreatic cancer has limited its application to treat the disease (Maxwell and Katz, 2021). Another limitation is that the organs adjacent to the pancreatic tumour, such as the stomach, duodenum and small bowel, are radiosensitive. Technological advances have improved the accuracy of targeting radiation to the tumour while sparing the surrounding healthy tissues, and stereotactic beam RT allows the delivery of high doses of radiation in a lower number of fractions due to a steep decrease in the radiation dose gradient at the edge of the target tissue. Initial studies in pancreatic cancer demonstrated local control with rare low-grade toxicity (Ng and Herman, 2018). Such advances may have the potential to increase the number of patients suitable for clinical trials of RT.

The lack of clinical trials of RT in pancreatic cancer may also be due in part to a lack of clinically relevant preclinical research (Ahmad et al., 2019) and a lack of understanding of the effect of radiation on the pancreatic tumour microenvironment. Improved pathological outcomes observed in some RT trials suggest that there may be at least a subgroup of patients who would benefit from neoadjuvant CRT; however, further investigation is needed to identify these patients and the mechanisms involved. The small animal radiotherapy research platform (SARRP) combines CT and X-ray irradiation for targeted RT, at clinically relevant doses, in small animals (Wong et al., 2008), and therefore represents an ideal tool for preclinical radiation research that simulates the clinical setting. However, mouse abdominal organs and pancreatic tumours have very similar radiodensity properties, which hinders tumour localisation and segmentation using CT. Therefore, we sought to optimise imaging using the built-in CT scanner of the SARRP to visualise abdominal anatomy in autochthonous models of pancreatic cancer and plan treatment studies in a relatively high-throughput manner. Iodine contrast has several advantages over alternative techniques such as the placement of a fiducial marker, which requires surgery or expertise in image-guided injections (Seifert et al., 2016). Magnetic resonance imaging has also been used successfully but this is time-consuming and may not be available to all research centres (Dobiasch et al., 2018). We further aimed to determine the efficacy of RT in autochthonous pancreatic cancer using the gold-standard *LSL-Kras*^G12D/+^; *LSL-Trp53*^R172H/+^; *Pdx1-Cre* (KPC) mouse model (Hingorani et al., 2005). We demonstrate that iodine contrast agents can enhance CT imaging to allow the localisation and safe targeting of autochthonous pancreatic tumours with RT and show the efficacy of different targeted irradiation regimens in pancreatic tumours in KPC mice.

## RESULTS

### Administration of iohexol for CT imaging of pancreatic tumours

Recently, irradiation platforms that simulate clinical conditions have been developed for use in preclinical research. The Xstrahl SARRP incorporates three-dimensional (3D) CT imaging and software to enable targeted dose delivery of radiation and clinically relevant treatment planning. Fields as small as 1×1 mm, combined with the ability to deliver the beam from any angle, allow for more precise tumour targeting, thus minimizing toxicity to surrounding tissue. In order to facilitate higher-throughput testing of preclinical RT in clinically relevant mouse models of pancreatic cancer, we first wanted to optimise a CT imaging protocol for the targeted irradiation of pancreatic tumours in KPC mice. KPC mice express pancreas-specific mutant *Kras*^G12D^ and *Trp53*^R172H^ and almost all mice spontaneously develop metastatic PDAC and succumb to disease between 70 and 300 days of age, with a median survival of ∼140 days (Hingorani et al., 2005). Localised PDAC, suitable for targeted RT, is detectable by manual palpation and ultrasound imaging from 70 days of age. The tumours that develop recapitulate many features of the human disease, namely, a fibrotic microenvironment rich in myeloid cells with a paucity of lymphocytes, metastasis to the liver and lungs, and resistance to conventional treatments. This therapeutic resistance makes the model well suited for preclinical trials of novel therapeutic regimens, including RT.

**Fig. 1.**
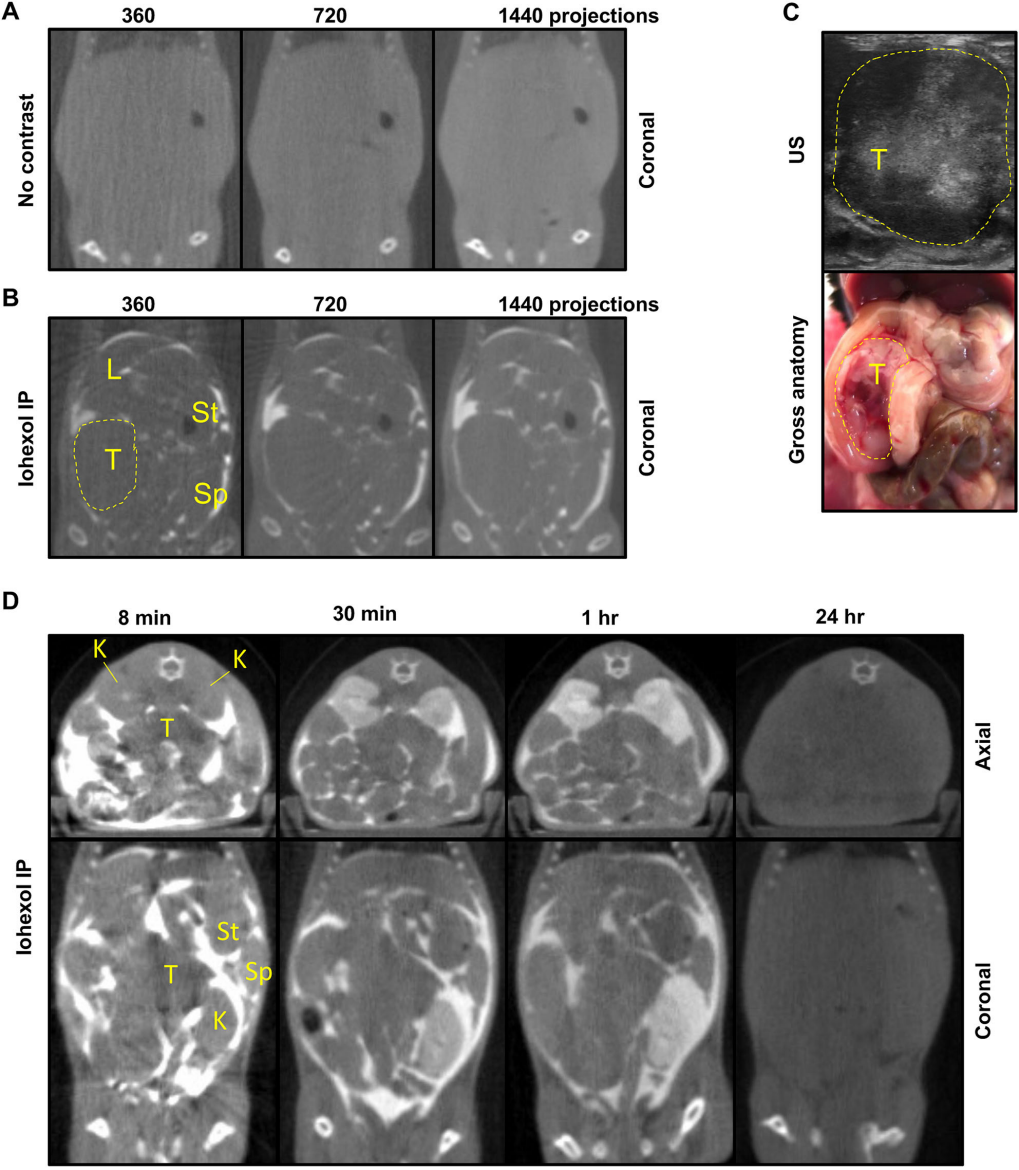
**Intraperitoneal administration of iohexol allows pancreatic tumour delineation by computed tomography imaging in the autochthonous KPC model.** (A) Representative examples of computed tomography (CT) scans, built from 360, 720 or 1440 projections as indicated, of a tumour-bearing KPC mouse without addition of contrast agent (*n*≥3 mice). (B) Representative examples of CT scans, built from 360, 720 or 1440 projections as indicated, of a tumour-bearing KPC mouse 8 min after intraperitoneal (IP) administration of 250 µl iohexol (*n*≥3 mice). The dashed line delineates the tumour. (C) Ultrasound (US) imaging (top panel) and gross anatomy (bottom panel) of the mouse from A,B. The dashed line delineates the tumour. (D) Representative examples of 1440 CT scans of a tumour-bearing KPC mouse at the indicated timepoints following IP administration of 250 µl iohexol (*n*≥2 mice). K, kidney; L, liver; Sp, spleen; St, stomach; T, tumour (pancreatic ductal adenocarcinoma or PDAC).

We first tested whether the in-built CT scanner within the SARRP enabled accurate delineation of autochthonous pancreatic tumours. Without contrast-enhancing methods, the abdominal tissues in the KPC mouse model were undistinguishable, thus precluding tumour delineation (Fig. 1A, left panel). To test whether increasing the number of projections acquired to build the CT image could enhance abdominal contrast, we increased the number of projections from 360 to 720 and 1440; however, this did not allow the delineation of abdominal tissues (Fig. 1A, middle and right panels). We then evaluated whether the radiopaque iodinated compound iohexol (Omnipaque™) could enhance abdominal contrast. Previous studies used 3 ml iohexol administered intraperitoneally to demarcate abdominal organs (Thorek et al., 2015). To refine this regimen, we tested whether a smaller volume could be used to outline pancreatic tumours in the KPC model. The outlines of abdominal organs and pancreatic tumours were visible on CT scans conducted 8 min following intraperitoneal (IP) injection of 250 µl of iohexol (Fig. 1B). An ultrasound scan and post-mortem dissection confirmed the presence of the pancreatic tumour in the location determined by contrast CT (Fig. 1C).

Next, we acquired CT scans at several timepoints up to 24 h following IP iohexol administration. The 8-min timepoint was the earliest selected owing to the time required from iohexol administration to acquisition of a 1440-projection CT scan. Imaging at this timepoint enabled accurate delineation of abdominal anatomy (Fig. 1D, left panel). At 30 min and 1 h post IP administration, we observed evidence of tissue absorption and excretion of iohexol, as indicated by increased renal contrast and lower contrast in the peritoneal space (Fig. 1D, middle panels). We observed complete clearance of iohexol 24 h post administration (Fig. 1D, right panel). These experiments demonstrated the requirement for iohexol administration immediately prior to CT scan acquisition.

We also tested alternative routes of iohexol administration as previously reported (Forsgard et al., 2016; Yahyanejad et al., 2015). We hypothesised that oral (*per os* or PO) administration could be used to highlight the duodenum and the stomach, which are radiosensitive organs in close proximity to pancreatic tumours. We could identify the duodenum and the stomach 8 min after PO administration of 250 µl iohexol in a tumour-bearing KPC mouse, demonstrating feasibility, but we could not delineate the tumour owing to the lack of contrast in the peritoneal cavity (Fig. S1A). Dual PO and IP administration of iohexol in the same mouse did not provide contrast benefit beyond that afforded by IP alone (Fig. S1B). We also tested whether intravenous (IV) administration of iohexol could directly enhance tumour contrast instead of outlining the tumour by IP injection; however, IV injection of 150 µl iohexol did not provide tumour contrast (Fig. S1C). Furthermore, iohexol was cleared from the bloodstream within minutes of IV administration as indicated by kidney contrast (Fig. S1C). Therefore, we sought to test alternative iodinated molecules able to directly bind to pancreatic tumours.

### Administration of Fenestra LC for CT imaging of pancreatic tumours

Fenestra LC is an iodinated triglyceride previously used as a liver contrast agent. It has been shown to be visible in the vasculature immediately after IV administration and to accumulate slowly in the liver over 24 h (Ford et al., 2006). We tested whether Fenestra LC could accumulate in pancreatic tumours and thus improve visualisation of PDAC by CT imaging. In KPC mice, in which the presence of PDAC had been confirmed by ultrasound imaging, the tumour outline was not clearly visible by CT scanning 1 h post IV administration of Fenestra LC, although major abdominal blood vessels were visible (Fig. S1D). At 24 h post IV administration, we observed contrast in the liver, the spleen and the pancreatic tumour (Fig. S1E, left panel). Post-mortem analysis confirmed a pancreatic tumour at the location determined by the CT scan (Fig. S1E, right panel), demonstrating successful localisation of pancreatic tumours by CT scans 24 h after IV administration of Fenestra LC. To confirm that Fenestra LC accumulates in pancreatic tumours in the mice, we performed CT imaging of abdominal organs *ex vivo*. We used the Fenestra LC-avid liver and spleen as positive controls and the kidney and lung as negative controls (Fig. S1F). Contrast intensity in the pancreatic tumour was heterogeneous, ranging from high uptake similar to that of the liver to low uptake similar to that of the kidney after 24 h (Fig. S1F), in keeping with the heterogeneity observed *in vivo* (Fig. S1E).

As Fenestra LC accumulates in pancreatic tumours, suggesting direct binding, we hypothesised that Fenestra LC could allow visualisation of pancreatic tumours for several days following a single administration, potentially facilitating imaging over several RT sessions without repeated administration of contrast agent. Therefore, we tested whether pancreatic tumours would still be detectable by CT scanning up to 6 days after Fenestra LC IV administration. At this timepoint, we were still able to detect the pancreatic tumour (Fig. S1G), although contrast was more marked in the tumour borders, again illustrating the heterogeneity of tumour binding.

Taken together, these data demonstrate the feasibility of using Fenestra LC to directly localise pancreatic tumours in KPC mice using the built-in CT scanner of the SARRP. Daily fractionation is possible for at least 6 days with a single administration of Fenestra LC, whereas administration of iohexol would be required prior to each fraction.

### Targeted irradiation of pancreatic tumours in KPC mice

The biological effect of radiation is oxidisation, which causes double-strand breaks in DNA. As a hallmark of the radiation response is the phosphorylation of histone H2A.X (γH2A.X) at the site of double-strand breaks, we used γH2A.X immunostaining to evaluate the accuracy of targeted RT in autochthonous pancreatic tumours localised using either iohexol or Fenestra LC contrast.

**Fig. 2.**
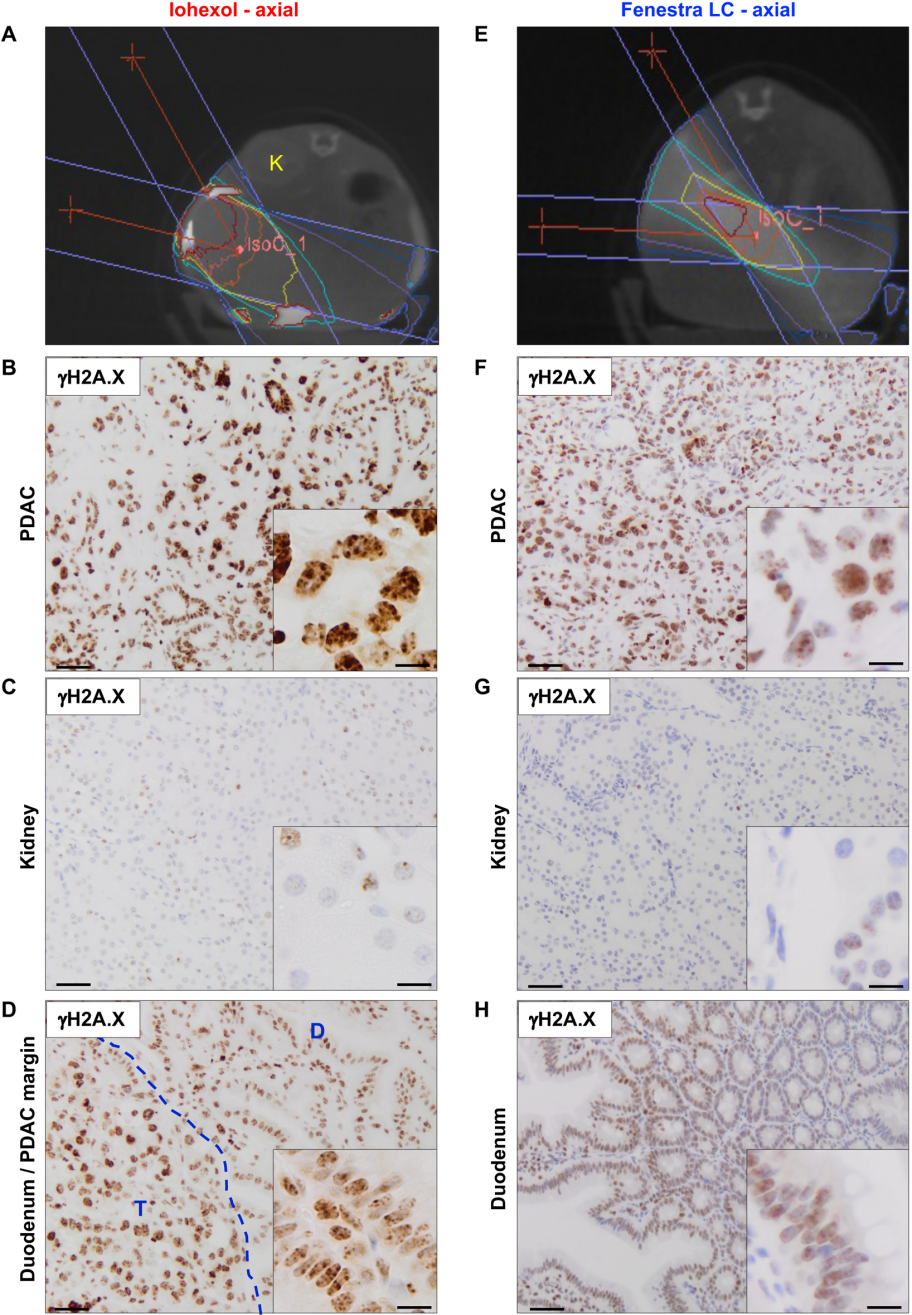
**Targeted irradiation of pancreatic tumours in the autochthonous KPC model.** (A) Representative radiation plan on a 1440-projection CT scan of a tumour-bearing KPC mouse immediately following IP injection of iohexol to provide tissue contrast (*n*=25 mice). In this animal, radiation planning was designed to spare the right kidney (K) adjacent to the tumour. (B-D) Representative images of immunohistochemistry for γH2A.X on (B) PDAC, (C) the right kidney and (D) the duodenum–PDAC margin showing γH2A.X staining in brown in the radiation path. Tissues were harvested from the KPC mouse shown in A 1 h after 4 Gy arc therapy delivered using a 10×10 mm collimator. Iohexol was administered intraperitoneally immediately prior to acquisition of the 1440-projection CT scan (*n*=4 mice). The dashed blue line in D indicates the border between tumour tissue and the adjacent duodenum. D, duodenum; K, kidney; T, tumour (PDAC). (E) Representative radiation plan on a 1440-projection CT scan of a tumour-bearing KPC mouse 24 h after intravenous (IV) injection of Fenestra LC to provide tissue contrast (*n*=5 mice). In this animal, radiation planning was designed to spare the left kidney on the opposite side of the beam. (F-H) Immunohistochemistry for γH2A.X on (F) PDAC, (G) the left kidney and (H) the duodenum showing γH2A.X staining in brown in the radiation path. Tissues were harvested from the KPC mouse shown in E 1 h after 4 Gy arc therapy delivered using a 5×5 mm collimator. Fenestra LC was administered intravenously 24 h prior to acquisition of the 1440-projection CT scan (*n*=1). Scale bars: 50 µm (main images); 10 µm (insets).

Iohexol was administered intraperitoneally for tumour localisation on a 1440-projection CT scan, and 4 Gy radiation was delivered by arc therapy to the tumour. Collimator size selection was based on tumour size to allow targeting of the whole tumour, whereas radiation planning was designed to avoid nearby organs (Fig. 2A). γH2A.X-positive nuclei were detected in the pancreatic tumour (Fig. 2B) but not in the adjacent kidney 1 h post irradiation (Fig. 2C), indicating successful targeting. The accuracy of targeted irradiation depends not only on the quality of imaging, but also on the relative position, shape and size of both the collimator and the tumour. Thus, irradiation of normal tissues directly adjacent to the tumour, such as the duodenum, is common. In keeping with this, γH2A.X immunostaining of the margin between the tumour and the duodenum also showed evidence of irradiation of the duodenum (Fig. 2D).

We next performed γH2A.X immunostaining on sections of tissues harvested 1 h post irradiation using Fenestra LC for imaging. The radiation plan was designed to deliver a dose of 4 Gy by arc therapy to the tumour, while avoiding nearby organs, and collimator size selection was again based on tumour size to allow targeting of the whole tumour (Fig. 2E). γH2A.X-positive nuclei were again detected in the pancreatic tumour (Fig. 2F) but not in the kidney (Fig. 2G) 1 h post irradiation, indicating successful targeting. In this case, irradiation of the duodenum was minimal (Fig. 2H), likely owing to both location and a smaller collimator size.

Taken together, these data demonstrate that CT imaging using either IP iohexol or IV Fenestra LC administration for tumour delineation provides sufficient contrast for successful targeted irradiation of pancreatic tumours in KPC mice.

### Safety of radiation delivery by SARRP

**Fig. 3.**
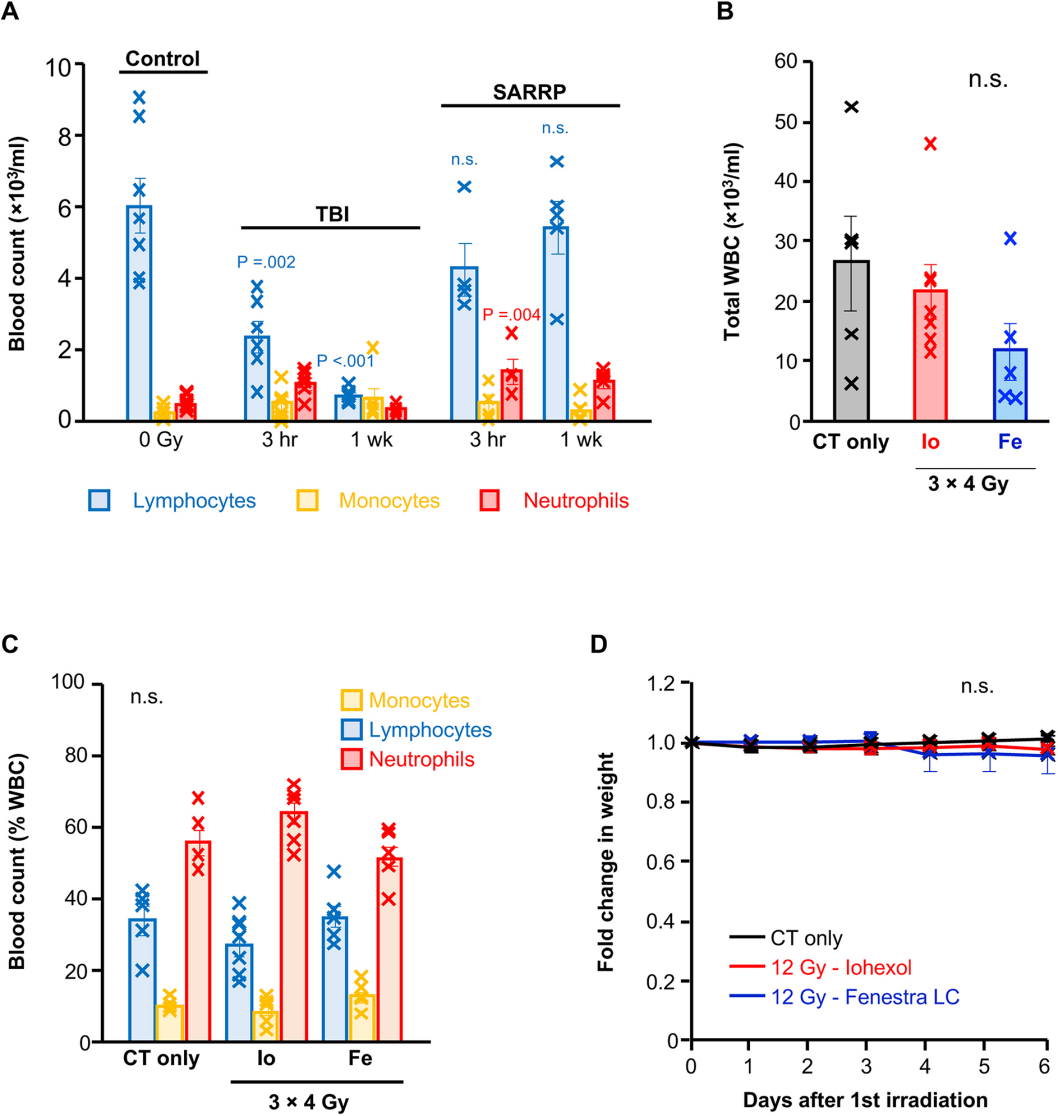
**Minimal toxicity associated with targeted irradiation by SARRP in the autochthonous KPC model**. (A) Leukocyte counts in the blood of wild-type mice 3 h or 1 week after 4 Gy radiation was delivered by total body irradiation (TBI) or targeted irradiation to abdominal soft tissues using the small animal radiotherapy research platform (SARRP). Control mice were unirradiated and not subject to CT acquisition (0 Gy, *n*=7 mice; TBI 3 h, *n*=6 mice; TBI 1 week, *n*=6 mice; SARRP 3 h, *n*=4 mice, SARRP 1 week, *n*=5 mice). Bars represent mean blood cell count±s.e.m. (B) Total white blood counts (WBC) in tumour-bearing KPC mice exposed to 18.66 cGy during CT acquisition or exposed to three fractions of 4 Gy targeted to the tumour using iohexol (Io) or Fenestra LC (Fe) contrast-enhanced CT scanning (CT only, *n*=5 mice; iohexol, *n*=7 mice; Fenestra LC, *n*=5 mice). Bars represent total WBC count±s.e.m. (C) Leukocyte proportions in the blood of tumour-bearing KPC mice exposed to 18.66 cGy during CT acquisition or exposed to three fractions of 4 Gy targeted to the tumour using iohexol or Fenestra LC contrast-enhanced CT scanning (CT only, *n*=5 mice; iohexol, *n*=7 mice; Fenestra LC, *n*=5 mice). Bars represent mean blood cell count±s.e.m. In A-C, each cross represents an individual mouse. Statistical significance was tested by one-way ANOVA with Bonferroni correction. (D) Fold change in animal weight during and after irradiation. CT-only controls received 18.66 cGy during CT acquisition. Irradiated mice received three fractions of 4 Gy on days 0, 2 and 4, targeted to the tumour using iohexol or Fenestra LC contrast-enhanced CT scanning (CT only, *n*=5 mice; iohexol, *n*=7 mice; Fenestra LC, *n*=3 mice). Crosses represent mean weight±s.e.m. per group. Statistical significance was tested by one-way ANOVA with Bonferroni correction at each timepoint. n.s., not significant, *P*>0.05.

The dose-limiting organ of total body irradiation (TBI) is the bone marrow. To test whether SARRP irradiation causes haematopoietic toxicity, we compared the effects of targeting 4 Gy of radiation to abdominal soft tissues of wild-type mice by SARRP with the effects of TBI. We observed that 4 Gy TBI reduced the number of circulating lymphocytes 3 h post irradiation and lymphocyte depletion was sustained for at least 1 week (Fig. 3A). In contrast, 4 Gy delivered by SARRP using a 5×5 mm collimator to abdominal soft tissues did not result in lymphopenia (Fig. 3A).

Next, we obtained blood counts from mice at endpoint that received three fractions of 4 Gy targeted to pancreatic tumours by SARRP using iohexol or Fenestra LC imaging contrast. We used mice that were CT scanned only as our comparator to control for the small amount of radiation (18.66 cGy) that the mice were exposed to during acquisition of the CT scan. There was no significant difference in the total numbers of circulating white blood cells (Fig. 3B) or in the proportions of circulating leukocytes (Fig. 3C). These observations suggested that SARRP irradiation does not cause haematopoietic toxicity and is suited for the investigation of combined radio-immunotherapy in KPC mice.

As tumour-targeted irradiation may also result in duodenal irradiation (Fig. 2), we monitored body weight as a measure of gut toxicity. There was no significant weight loss throughout the course of RT (Fig. 3D), indicating normal intestinal function.

### Efficacy of radiation delivery by SARRP

We next wanted to investigate the efficacy of different targeted RT regimens, delivered by SARRP, in KPC mice. We first confirmed that there was no significant difference in survival of mice subjected to radiation through CT acquisitions only (18.66 cGy) compared to that of unirradiated mice (Fig. S2A). Furthermore, there was no difference in tumour growth rate (Fig. S2B), enabling pooling of unirradiated mice and mice that were subjected to CT acquisition only as our control group. Neither did the use of contrast agents influence survival or tumour growth rate following three fractions of 4 Gy (Fig. S2A,C). Therefore, mice irradiated using either iohexol or Fenestra LC were included in our RT cohorts.

**Fig. 4.**
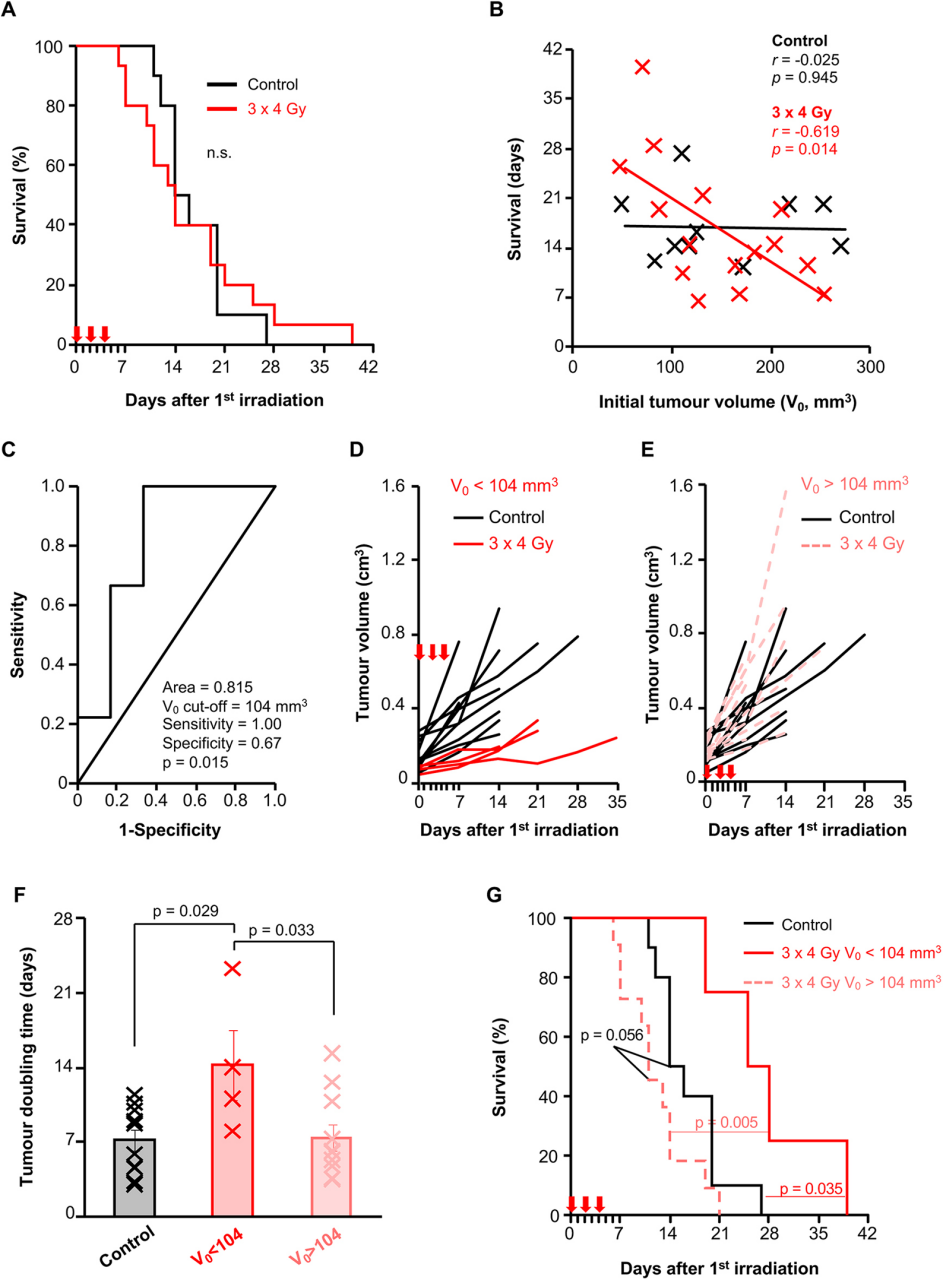
**Response to three fractions of 4 Gy SARRP irradiation in the autochthonous KPC model.** (A) Kaplan–Meier survival analysis of tumour-bearing KPC mice irradiated with three fractions of 4 Gy (3×4 Gy) on days 0, 2 and 4 (red arrows) by SARRP (red line; *n*=15 mice; median=16.3 days), or unirradiated/only exposed to 18.66 cGy during CT acquisition (black line; *n*=10 mice; median=16.8 days). Statistical significance was tested using the log-rank test. n.s., not significant, *P*>0.05. (B) Scatter plot showing the correlation between survival and initial tumour volume (V_0_) in the control or 3×4 Gy-irradiated mice shown in A. *r*, Pearson correlation coefficient. (C) ROC curve analysis to define the most powerful initial tumour volume cut-off (V_0_=104 mm^3^) that distinguishes differences in survival in the tumour-bearing KPC mice shown in A. The mean survival of mice irradiated with 3×4 Gy was used to categorise short- and long-term survivors. (D) Tumour volume monitoring by weekly 3D ultrasound scanning of control KPC mice (black lines, *n*=10 mice) or KPC mice irradiated with 3×4 Gy on days 0, 2 and 4 (red arrows) with an initial tumour volume of <104 mm^3^ (red lines, *n*=4 mice). Each mouse is plotted individually. (E) Tumour volume monitoring by weekly 3D ultrasound scanning of control KPC mice (black lines, *n*=10 mice, data from the same control mice in D) or KPC mice irradiated with 3×4 Gy on days 0, 2 and 4 (red arrows) with an initial tumour volume of >104 mm^3^ (red dotted lines, *n*=11 mice). Each mouse is plotted individually. (F) Chart showing tumour-doubling time in control KPC mice (*n*=10 mice) or KPC mice irradiated with 3×4 Gy and with an initial tumour volume of <104 mm^3^ (*n*=4 mice) or >104 mm^3^ (*n*=11 mice). Each cross represents an individual mouse. The bars represent mean tumour-doubling time±s.e.m. Statistical significance was tested using one-way ANOVA with Bonferroni correction. (G) Kaplan–Meier survival analysis of control KPC mice (*n*=10 mice, data from the same control mice in 4A) or KPC mice irradiated with 3×4 Gy and with an initial tumour volume of <104 mm^3^ (*n*=4 mice) or >104 mm^3^ (*n*=11 mice). Statistical significance was tested in pairwise comparisons using the log-rank test.

**Fig. 5.**
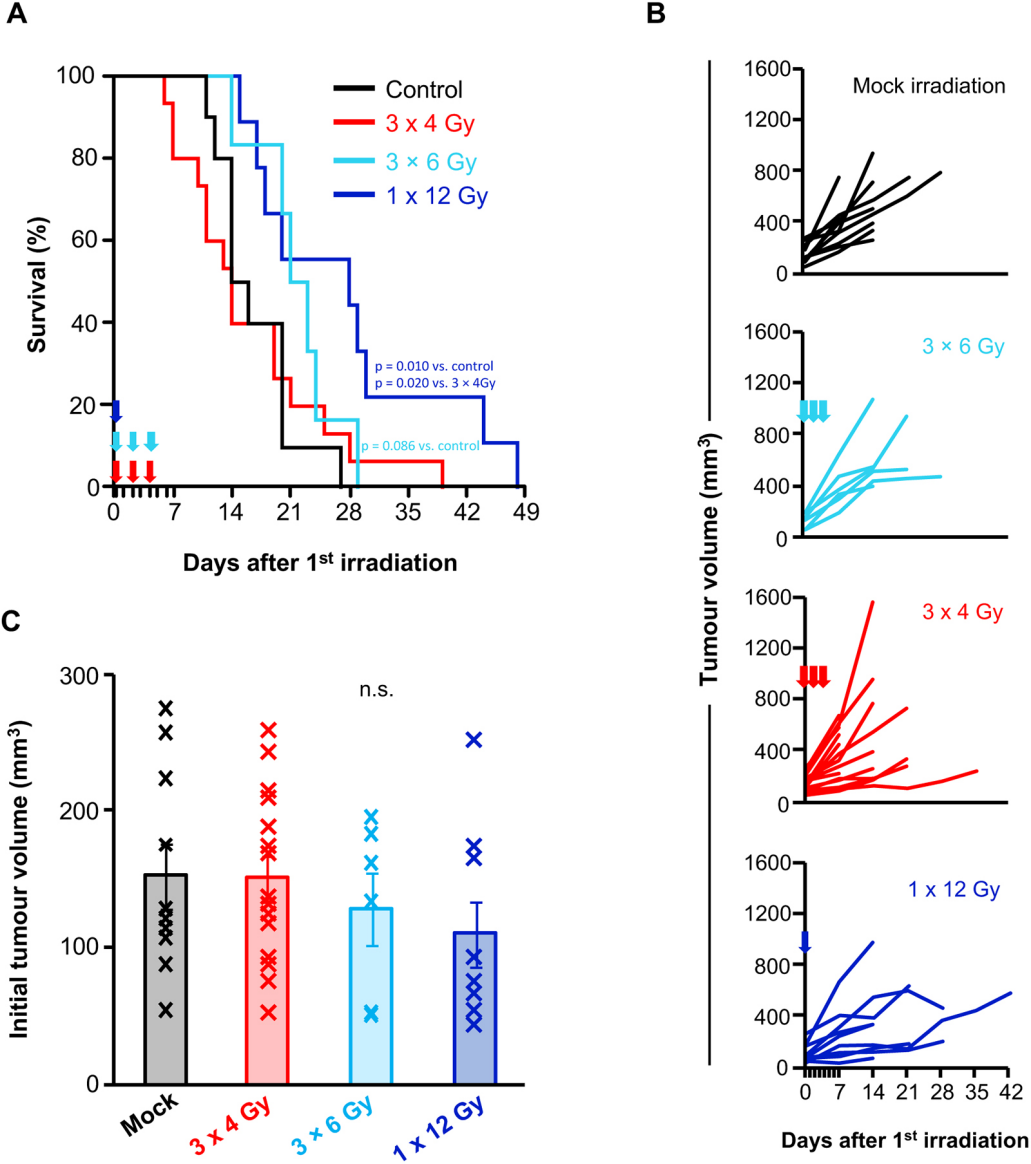
**Efficacy of increased dose or hypofractionation of irradiation in the autochthonous KPC model.** (A) Kaplan–Meier survival analysis of control KPC mice (black line, *n*=10 mice, median=16.8 days; data from the same control mice in Fig. 4A) or KPC mice irradiated with three fractions of 4 Gy (3×4 Gy) on days 0, 2 and 4 (red arrows; red line, *n*=15 mice, median=16.3 days; data from the same mice irradiated with 3×4 Gy in Fig. 4A), three fractions of 6 Gy (3×6 Gy) on days 0, 2 and 4 (cyan arrows; cyan line, *n*=6 mice, median=21.8 days) or one fraction of 12 Gy (1×12 Gy) on day 0 (blue arrow; blue line, *n*=9 mice, median=27.7 days). Statistical significance was tested in pairwise comparisons using the log-rank test. (B) Tumour volume monitoring by weekly 3D ultrasound scanning of control KPC mice (mock irradiation, *n*=10 mice) or KPC mice irradiated with 3×4 Gy (red, *n*=15 mice), 3×6 Gy (cyan, *n*=6 mice) or 1×12 Gy (blue, *n*=9 mice). Each mouse is plotted individually. Data from the control and 3×4 Gy mice are the same as in Fig. 4D,E. (C) Chart showing initial tumour volume in control KPC mice (*n*=10 mice) or KPC mice irradiated with 3×4 Gy (*n*=15 mice), 3×6 Gy (*n*=6 mice) or 1×12 Gy (*n*=9 mice). Each cross represents an individual mouse. The bars represent mean initial tumour volume±s.e.m. Statistical significance was tested using one-way ANOVA with Bonferroni correction. n.s., not significant, *P*>0.05.

We found that three fractions of 4 Gy did not prolong survival in tumour-bearing KPC mice (Fig. 4A); however, we did notice a tail of longer-term survivors. Therefore, we tested whether the initial tumour volume could influence the therapeutic efficacy of RT in this model. We found a significant inverse correlation between survival and initial tumour volume in the radiation group but not in the control group (Fig. 4B). Receiver operator curve (ROC) analysis indicated that an initial tumour volume of V_0_=104 mm^3^ was the most appropriate cut-off to predict survival benefit (Fig. 4C). We then compared RT efficacy in mice bearing smaller (V_0_<104 mm^3^) and larger (V_0_>104 mm^3^) tumours prior to treatment. Three fractions of 4 Gy delayed growth of smaller tumours (Fig. 4D) but not of larger tumours (Fig. 4E), as indicated by a significant increase in tumour-doubling time only in irradiated mice with an initial tumour volume<104 mm^3^ (Fig. 4F). This was associated with a significant survival advantage in mice bearing smaller tumours compared to those with larger tumours (Fig. 4G). We found no significant difference in the percentage of γH2A.X-positive cells between smaller and larger irradiated tumours, suggesting similar DNA damage repair capacity (Fig. S3). We next assessed whether increased dose or hypofractionation had any impact on therapeutic efficacy. We found that delivering three fractions of 6 Gy (*P*=0.086) or one fraction of 12 Gy (*P*=0.010) radiation both extended survival in tumour-bearing KPC mice compared to that in control mice (Fig. 5A), and this was also associated with reduced tumour growth in mice irradiated with one fraction of 12 Gy radiation as monitored by ultrasound imaging (Fig. 5B). Importantly, initial tumour volumes were comparable across cohorts (Fig. 5C). Thus, our data show that efficacy is increased with higher dose or hypofractionation and suggest that exploration of further dosing strategies is warranted.

**Fig. 6.**
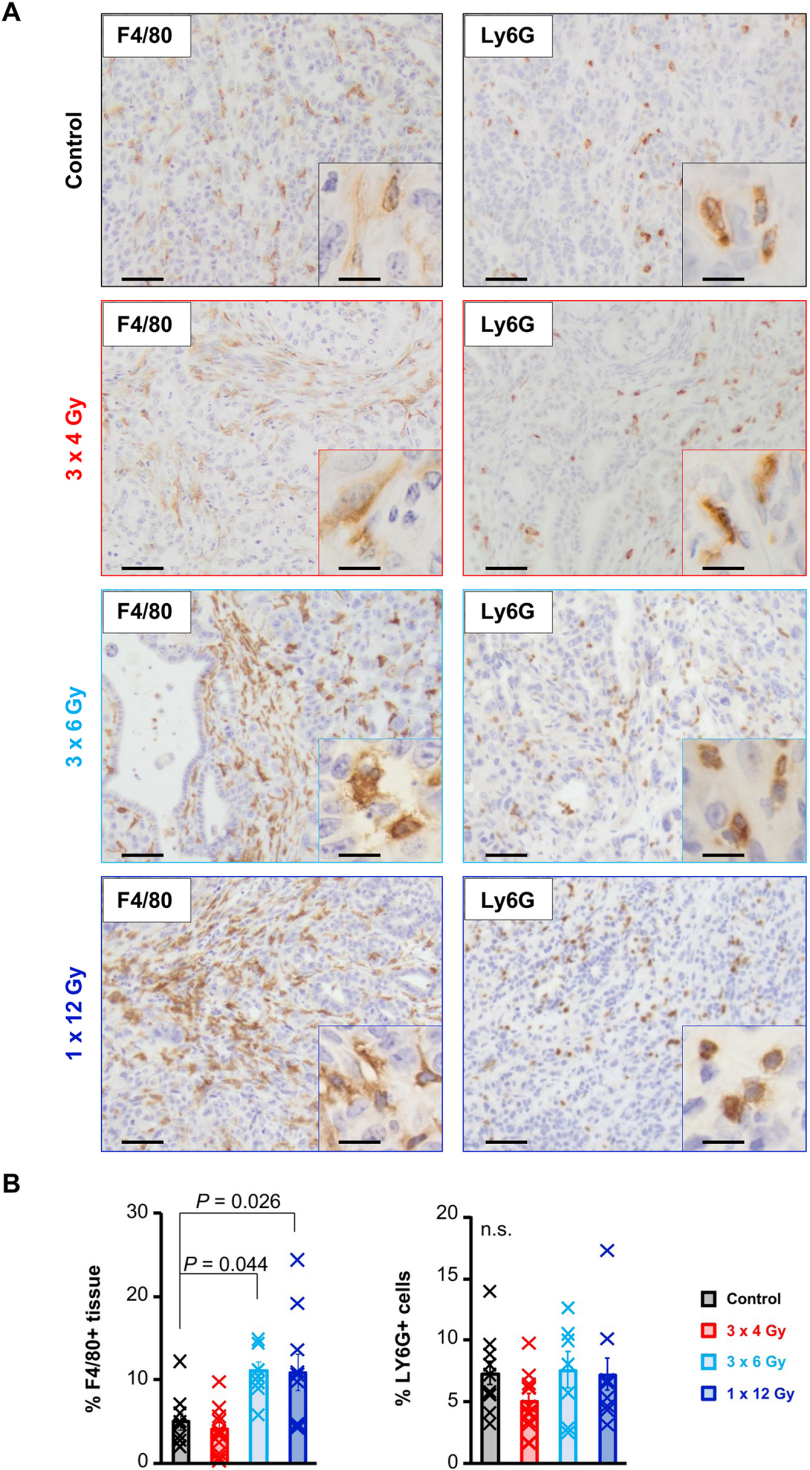
**The myeloid content of SARRP-irradiated pancreatic tumours in KPC mice.** (A) Representative images of immunohistochemical staining for F4/80 (macrophages) and Ly6G (neutrophils) in PDAC harvested at endpoint from control KPC mice (*n*=10) or from KPC mice irradiated with three fractions of 4 Gy on days 0, 2 and 4 (red, *n*=12), three fractions of 6 Gy on days 0, 2 and 4 (cyan, *n*=7) or one fraction of 12 Gy on day 0 (blue, *n*=10). Immunopositivity is brown and nuclei are counterstained in blue. Scale bars: 50 µm (main images); 10 µm (insets). (B) HALO software was used to quantify macrophages and neutrophils in PDAC sections from the mice described in A. Each cross represents an individual mouse. Bars represent mean score±s.e.m. Statistical significance was tested using one-way ANOVA with Bonferroni correction. n.s., not significant, *P*>0.05.

Our ability to safely irradiate pancreatic tumours in an autochthonous model provided an opportunity to improve our understanding of the effect of radiation on the pancreatic tumour microenvironment. Macrophages have previously been shown to respond to radiation and influence cancer outcomes (Beach et al., 2022), and PDAC is characterised by extensive myeloid cell accumulation. Thus, we first investigated how RT affected myeloid cell populations in PDAC in our model. Immunohistochemistry for the macrophage marker F4/80 (ADGRE1) revealed a significant increase in the number of intratumoural macrophages following irradiation with three fractions of 6 Gy or one fraction of 12 Gy, but not with lower dose RT (Fig. 6A,B). In contrast, we did not observe any changes in the number of tumour-infiltrating neutrophils (Fig. 6A,B). Furthermore, although RT has been suggested to elicit an enhanced adaptive immune response, we found no difference in the number of intratumoural lymphocytes following tumour-targeted irradiation (Fig. S4). Similarly, cancer-associated fibroblasts (CAFs) can alter their secretory output in response to RT, thus impacting the tumour microenvironment (Ansems and Span, 2020). Hence, we examined the stromal response in tumours in KPC mice following RT. Immunohistochemistry for the pan-CAF marker, podoplanin (PDPN), revealed no significant increase in the number of CAFs in tumours following irradiation, which was consistent with similar levels of collagen deposition in irradiated KPC tumours compared with those in KPC control tumours (Fig. S5).

Finally, as we observed greater radiotherapeutic efficacy with three fractions of 4 Gy in smaller versus larger tumours (Fig. 4), we wondered whether tumour size at the time of treatment might impact radiation-induced changes in the composition of the tumour microenvironment. However, we found no difference in the number of tumour-associated macrophages, neutrophils, T cells, B cells or fibroblasts, or in the amount of collagen, between smaller and larger tumours (Fig. S6), suggesting that initial tumour volume does not influence longer-term tumour microenvironment response to RT.

Taken together, our data demonstrate the safety and feasibility of delivering a targeted therapeutic dose of radiation in pancreatic tumour-bearing mice to study the radiation response *in vivo* and to monitor disease progression. Our method allows for high-throughput preclinical testing of RT strategies in autochthonous mouse models of pancreatic cancer with a clinically relevant microenvironment.

## DISCUSSION

Recent advances in cancer genomics and transcriptomics have driven increased interest in trials investigating personalised therapy in cancer, including pancreatic cancer. However, despite some promising preclinical studies examining chemoradiation strategies, there has been a failure to translate novel RT-based approaches to the clinic (Ahmad et al., 2019). One obvious requirement to progress promising chemoradiation strategies into the clinic is robust data showing both tolerability and efficacy in clinically relevant models (Ahmad et al., 2019). Here, we provide data to support and facilitate the use of the autochthonous KPC mouse model, which recapitulates many features of the human disease, for preclinical radiation research in pancreatic cancer. We have shown the feasibility and safety of using iodine contrast to localise pancreatic tumours, target radiation to the tumour using the SARRP system, evaluate the radiation response and monitor disease progression in a relatively high-throughput manner.

We refined a method for iohexol administration and successfully repurposed the liver imaging agent Fenestra LC to localise and irradiate pancreatic tumours. Both iodinated contrast agents have advantages and limitations. Iohexol is cheap and easy to use as it can be administered intraperitoneally, with minimal animal handling required, immediately before irradiation. However, prior knowledge of tumour location by ultrasound imaging is required to aid tumour localisation, and administration prior to each fraction is required, as iohexol is cleared from the peritoneal space within 24 h. On the other hand, Fenestra LC provides contrast in the pancreatic tumour, the liver and the spleen, rendering them readily distinguishable by shape and location without the need for prior imaging. In addition, accumulation within the tumour means that a single administration of Fenestra LC is sufficient for CT imaging over at least 6 days; however, Fenestra LC is more expensive and IV injection more invasive.

Our establishment of a robust platform for preclinical radiation oncology in autochthonous pancreatic cancer models allowed us to examine the efficacy of various fractionation regimens. We found that one fraction of 12 Gy in KPC mice provides better local tumour control than three fractions of 4 Gy, supporting the use of hypofractionated RT regimens in pancreatic cancer. Indeed, several trials of hypofractionated RT in pancreatic cancer are ongoing, typically examining one to five fractions of 5-20 Gy (Malla et al., 2022). The ability to conduct similar trials in mice in a relatively high-throughput manner should facilitate more efficient preclinical testing of radiosensitising strategies to provide proof of concept for clinical translation.

The main effect of tissue irradiation is the oxidation of biological molecules. Using drugs to increase the radiation-induced DNA damage burden in tumour cells has long been shown to be an effective radiosensitising strategy (Barker et al., 2015). However, tumour cell DNA is not the only irradiated biological molecule. Fibrosis has long been described in the chronic radiation response and, more recently, the effects of radiation on intratumoural endothelial, stromal and immune cells have been implicated in the tumour radiation response (Byrne et al., 2021). Pancreatic tumours are characterised by a strong desmoplastic microenvironment that can limit treatment efficacy (Pickup et al., 2014). Therefore, preclinical investigations of chemoradiation strategies must be designed to not only consider the DNA damage response in tumour cells, but also the changes in signalling in endothelial, stromal and immune cells in response to tumour irradiation. Tumours in the KPC model recapitulate the complex tumour microenvironment of pancreatic cancer and thus represent an ideal model in which to study the tumour radiation response.

In our study, we observed an increase in tumour-associated macrophages in PDAC following three fractions of 6 Gy or one fraction of 12 Gy. This suggested the involvement of macrophages in the *in vivo* radiation response of KPC tumours. Macrophages are highly plastic cells able to respond to microenvironmental cues, and radiation can affect macrophage polarisation and tumour recruitment in a context-dependent manner (Beach et al., 2022). Thus, although increased intratumoural macrophages correlate with good prognosis in our study, this increase may represent a mechanism of radioresistance that could be targetable with macrophage-targeting therapies. Although we did not observe significant changes in numbers of other immune or stromal cells in pancreatic tumours following radiation, this increase in macrophages could also be mediated by altered behaviour of other cells in the microenvironment in response to radiation. Thus, changes in the phenotype, rather than the number of these cell types may play a role in the response to radiation.

Interestingly, we found that exposure to three fractions of 4 Gy conferred some therapeutic benefit, but only in mice with smaller tumours at the time of irradiation. Importantly, there was no correlation between initial tumour volume and survival in control mice, reflecting the complex nature of PDAC as survival can be influenced by the location of the tumour, stromal content or the presence of metastases, ascites or cachexia. With regard to efficacy in smaller tumours, there are various possible explanations. First, the shape of the collimator and the tumour do not match perfectly, but the ability to increase the size of the irradiated field is limited by the radiosensitive tissues surrounding the tumour. Therefore, the entire tumour may not receive optimal radiation dosing. However, the percentage of tumour irradiated with a sub-therapeutic dose was similar in small and large tumours in our study. A second possibility is that larger tumours are more likely to contain larger hypoxic areas, a known contributing factor to radioresistance (Carlson et al., 2011). Third, although we found no difference in the ratio of γH2A.X-positive cells in tumours at endpoint, it is still possible that small tumours could have a greater DNA damage repair capacity in response to RT initially, which may no longer be observed at endpoint. Finally, the microenvironment may vary in larger versus smaller tumours and any differences could also promote radioresistance. We did not observe any differences in the composition of the microenvironment of tumours at endpoint between those that were smaller versus those that were larger at the time of irradiation. However, radiation insults may transiently affect the tumour microenvironment with potential effects on outcomes. Further studies into the temporal changes in the microenvironment and potential crosstalk among different cell types in response to radiation would increase our understanding of these effects.

RT efficacy in pancreatic cancer may be improved by using biomarkers of radiosensitivity to select the patients most likely to derive benefit. Based on the *LSL-Kras*^*G12D/+*^*; Pdx1-Cre* (KC) or KPC models, further models of pancreatic cancer have been developed that recapitulate some of the genetic heterogeneity observed in the human disease. These models would allow the investigation of the heterogeneity of the radiation response in pancreatic cancer harbouring different mutations and facilitate development of biomarkers of pancreatic tumour radiosensitivity. Mechanisms of radioresistance could also be studied in these models to develop precision radiosensitising strategies. Furthermore, RT has been implicated in promoting the invasive and metastatic potential of tumour cells (Chen et al., 2018; Li et al., 2016; Mori et al., 2021; Mueller et al., 2021; Yao et al., 2011), and RT in autochthonous models would enable exploration of this phenomenon *in vivo*. Indeed, although our study was not powered to answer this question, we did observe increased metastatic burden in irradiated mice. Ultimately the SARRP protocol described in this study will enable testing of RT responses in animal models recapitulating the heterogeneity of pancreatic cancer and allow investigation of the efficacy of chemoradiation regimens to inform clinical trials of precision RT in pancreatic cancer.

## MATERIALS AND METHODS

### Animals

All animal experiments were performed under a UK Home Office licence and approved by the University of Glasgow Animal Welfare and Ethical Review Board. Experimental mice were housed in conventional environmentally enriched cages and given access to standard diet and water *ad libitum*. *Pdx1-Cre*; *LSL-Kras*^G12D/+^; *LSL-Trp53*^R172H/+^ (KPC) mice (Hingorani et al., 2005), were bred in house at the CRUK Beatson Institute and maintained on a mixed background in individually ventilated cages. Mice of both sexes, in roughly equal proportions, were used in all cohorts. Where possible, single housing of mice was avoided. Mice were genotyped by Transnetyx (Cordoba, TN, USA). Mice were monitored at least three times a week and palpated weekly to detect pancreatic tumours. Mice were randomly assigned to treatment groups once tumours were confirmed by imaging, and follow-up scans were performed weekly until endpoint was reached. All mice on treatment were housed in the same location. Mice were humanely culled at endpoint when exhibiting moderate clinical signs of pancreatic cancer (swollen abdomen, loss of body conditioning resembling cachexia, reduced mobility). Animals that succumbed to extra-pancreatic pathologies were excluded. Post-mortem tumour burden was assessed by gross pathology, and organs were removed and fixed in 10% buffered formalin.

### CT imaging

Anaesthesia was induced and maintained throughout the procedure with a mixture of isoflurane and medical air. Abdominal anatomy of anaesthetized mice was imaged using the in-built cone beam CT (CBCT) function of the SARRP. Images were reconstructed using the Feldkamp, Davis and Kress CBCT reconstruction algorithm (Feldkamp et al., 1984) from 360, 720 or 1440 projections taken at 60 kV and 0.8 mA using the fine focal spot (1 mm). The absorbed dose associated with each CBCT was determined using an ionisation chamber and was corrected for temperature and pressure. Acquisition of a CT scan with 360, 720 or 1440 projections resulted in absorbed doses of 1.68, 3.19 or 6.22 cGy, respectively. To enhance contrast, we tested the tri-iodinated molecule iohexol {Omnipaque™, N,N′-bis(2,3-dihydroxypropyl)-5-[N-(2,3-dihydroxypropyl)-acetamido]-2,4,6 triiodoisophthalamide; GE Healthcare, UK} or the poly-iodinated emulsion Fenestra LC {1,3-bis-[7-(3-amino-2,4,6-triiodophenyl)-heptanoyl]-2-oleoyl glycerol; MediLumine, Bartec Technologies, UK} (Table S1).

### SARRP RT

Anaesthesia was induced and maintained throughout the procedure with a mixture of isoflurane and medical air. Anaesthetized mice were irradiated using the SARRP developed by Xstrahl. A 220 kV, 13 mA X-ray beam was used with a dose rate of approximately 280 cGy/min. Muriplan, the integrated preclinical treatment planning software, was used to segment the tissue, select the isocentre and precisely target and plan the irradiation of the tumour. Treatment was delivered by arc therapy. The broad focal spot (5.5 mm) was used and the collimator aperture size was selected *ad hoc* based on tumour size and shape. For immunostaining, a single dose of 4 Gy was delivered and the mice were euthanized 1 h post irradiation. For the treatment efficacy study, mice were irradiated with 12 Gy delivered in one fraction of 12 Gy or in three fractions of 4 Gy every other day, or with 18 Gy delivered in three fractions of 6 Gy every other day. The mock-irradiated control protocol consisted of three CT scans with 1440 projections resulting in 18.66 cGy TBI. Mice were euthanized when exhibiting moderate clinical signs of pancreatic cancer (as described above). Treatment efficacy was determined by Kaplan–Meier survival analysis with the log-rank test and by tumour size monitoring using 3D ultrasound imaging.

### TBI

TBI was performed using the RS225 irradiator (Xstrahl). Mice were placed in a mouse pie cage located 30 cm from the beam. A 195 kV, 10 mA X-ray beam was used with a dose rate of 160 cGy/min.

### Ultrasound imaging

For study enrolment and weekly on-treatment tumour-size monitoring, high-resolution ultrasound imaging was performed using the Vevo3100 system (FUJIFILM VisualSonics, Toronto, Ontario, Canada). The fur on the abdomen of mice was removed with depilatory cream prior to imaging. Anaesthesia was induced and maintained throughout the procedure with a mixture of isoflurane and medical air. The MX550D transducer was used to acquire 3D scans with a maximum depth of 15 mm and at 40 MHz. The transducer was controlled by a 3D motor, which maintained slow and incremental movement of the transducer to obtain 200-300 frames with a step size of 0.076 mm. Vevo LAB 3.1.1. software (VisualSonics) was used for tumour border annotation and 3D reconstruction of stacked images for tumour volume measurement.

### Histology and immunohistochemistry

Formalin-fixed tissues were paraffin embedded and 4 μm sections were acquired using a HistoCore MULTICUT microtome (Leica, UK). The sections were placed on poly-L-lysine slides and oven baked at 60°C for 2 h prior to Haematoxylin and Eosin (H&E) staining and immunohistochemistry. H&E staining was performed on a Leica autostainer (ST5020). Sections were dewaxed in xylene, rehydrated through graded alcohols and then stained with Haematoxylin Z (CellPath, UK). Sections were washed in water, immersed in 1% acid alcohol, washed again, and the nuclei ‘blued’ in Scott's tap water substitute (Dako, UK). Following washing, the sections were incubated in Putt's Eosin (Merck, UK) for ∼3 min. Picrosirius Red (PSR) staining was performed manually on sections dewaxed and rehydrated as above. Tissue sections were then stained for 2 h in PSR staining solution [50:50 volumes of 0.1% Direct Red 80 (Sigma-Aldrich) and 0.1% Fast Green (Raymond A. Lamb)] in distilled water diluted 1:9 with aqueous picric acid solution (VWR). Following staining, sections were rinsed in tap water, dehydrated through graded alcohols and cleared in xylene. Coverslips were placed on stained sections using DPX mountant (SEA-1300-00A, CellPath).

Immunohistochemistry was performed on formalin-fixed, paraffin-embedded tissue using standard protocols. Briefly, 4 μm sections were cut by microtome and placed on slides. Dewaxing was performed in xylene and the tissue was rehydrated by immersion through a series of graded alcohols. Peroxidase-blocking solution (Leica, UK) was used to quench endogenous peroxidase activity. Heat-induced epitope retrieval in citrate buffer (pH 6) was performed using a pre-treatment module (Dako, UK). The sections were washed in TBS containing 0.1% Tween 20 (TBST) before exposure to primary antibodies (Table S2). Slides were then incubated in secondary antibodies appropriate for the species (Dako EnVision, UK) for 30 min and then washed in TBST. 3,3′-diaminobenzidine tetrahydrochloride (Leica, UK) was applied for 0.5-10 min to visualise staining and the reaction was terminated by immersion in deionized water. Sections were counterstained with Haematoxylin and nuclei ‘blued’ in Scott's tap water. Finally, sections were then dehydrated through a graded alcohol series, cleared in xylene and mounted in DPX mountant (CellPath) under a glass coverslip. Automated scoring of staining on whole sections was performed using the HALO image analysis platform (Indica Labs).

### Haematology

At sacrifice, terminal blood samples were taken via cardiac puncture and transferred to EDTA-coated tubes. Blood counts were obtained using the ProCyte Dx Haematology Analyser (IDEXX).

### Statistics

Statistical analyses were carried out using SPSS software (IBM). Pearson's correlation coefficient was used to measure correlation. Receiver operator curve (ROC) analysis was used to determine cut-off values. Kaplan–Meier survival curves were analysed by the log-rank test. An independent-sample unpaired two-tailed *t*-test was used to detect significance and one-way ANOVA with Bonferroni correction was used for multiple comparisons.

## Supplementary Material

10.1242/dmm.050463_sup1 Supplementary information
