# Supplementary material for: Targeted irradiation in an autochthonous mouse model of pancreatic cancer
Source: Dis Model Mech. 2024 Mar 14;17(3):dmm050463. doi: 10.1242/dmm.050463 (PMC10958199; doi:10.1242/dmm.050463)
Supplement: Supplementary information [file dmm-17-050463-s1.pdf]

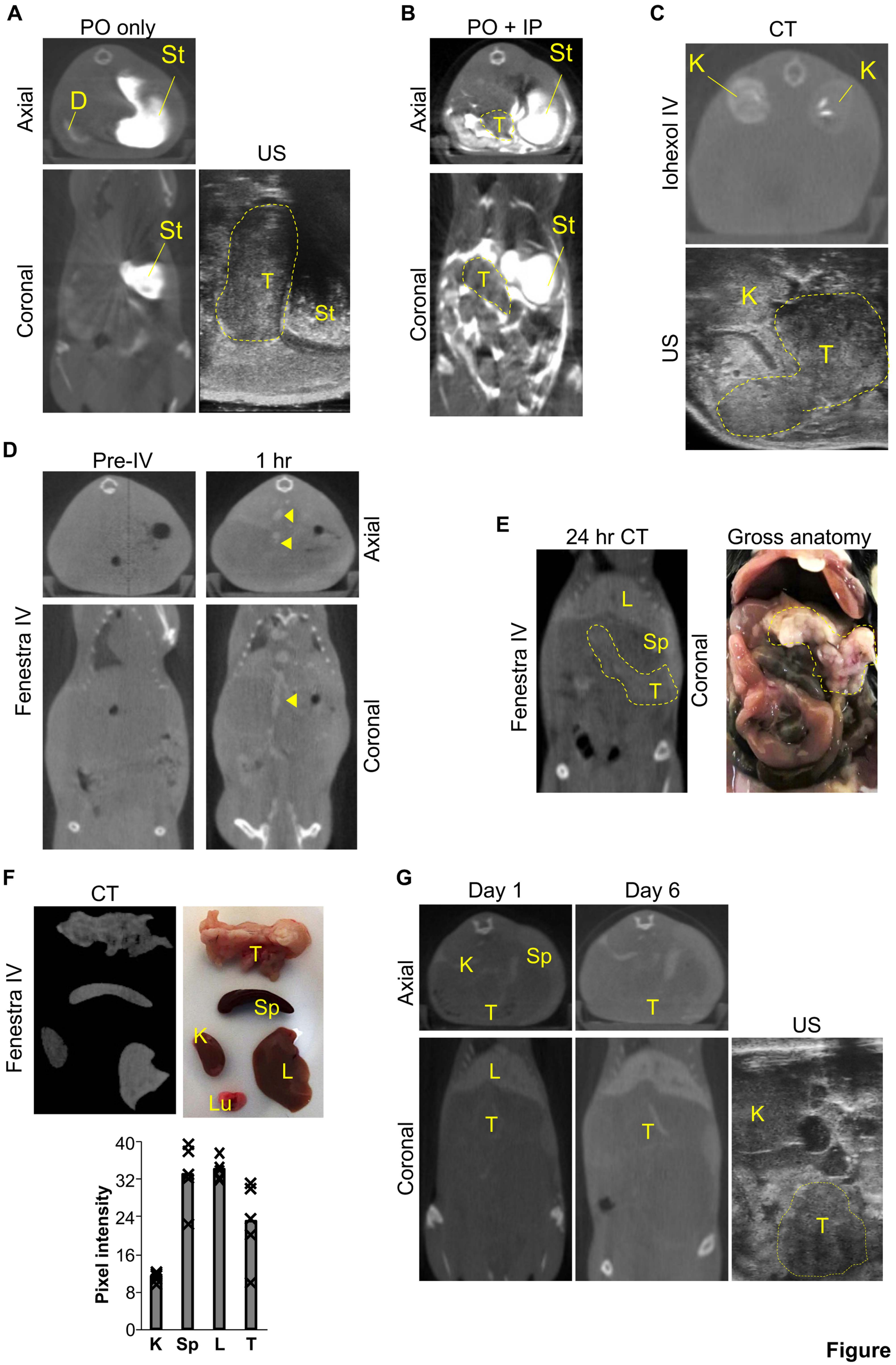

Figure S1

Disease Models & Mechanisms • Supplementary information

Fig. S1. Intravenous administration of Fenestra LC allows tumour delineation by CT imaging.

A) Representative example of a 1440 projection CT scan of a KPC mouse immediately after PO administration of 250 μl iohexol (n = 3 mice, left panel). The presence of a tumour in the same animal was confirmed by ultrasound (US) imaging, as highlighted by the dashed yellow line adjacent to the stomach (right panel).

B) Representative example of a 1440 projection CT scan of a KPC mouse immediately after dual PO administration of 250 μl iohexol and IP administration of 250 μl iohexol (n = 3 mice). The representative CT scan was obtained using the same tumour-bearing KPC mouse as in Figure S1A.

C) Representative example of a 1440 projection CT scan of a tumour-bearing KPC mouse 8 min after intravenous (IV) administration of 150 μl iohexol (n = 3 mice, top panel). The presence of a tumour in the same animal was confirmed by US imaging (bottom panel). The dashed line delineates the tumour only visible by US imaging.

D) Representative images of 1440 projection CT scans acquired prior to IV administration of Fenestra LC and 1 hr after in a tumour-bearing KPC mouse. Arrowheads indicate the location of Fenestra LC contrast in major abdominal blood vessels.

E) Representative image of a 1440 projection CT scan (left panel) acquired 24 hr after IV administration of Fenestra LC, which allowed *in situ* visualisation of the pancreatic tumour (n = 6 mice). The dashed line delineates the tumour. The presence of a pancreatic tumour was confirmed by post-mortem examination (right panel).

F) 1440 projection CT scan of the excised organs from the animal in Figure S1E (top left panel). The lung tissue could not be kept in focus with all other organs and was removed from densitometric analysis. Densitometric analysis of pixel intensity was performed using ImageJ. Histogram bars represent the mean pixel intensity of five random small areas within each tissue (bottom panel).

G) 1440 projection CT scans acquired 24 hr (left panels) and 6 days (middle panels) after IV administration of Fenestra LC. An ultrasound scan (right panel) prior to CT imaging confirmed the presence of a tumour below the kidney.

D, duodenum; St, stomach; K, kidney; T, tumour; L, liver; Sp, spleen; Lu, lung.

Disease Models & Mechanisms • Supplementary information

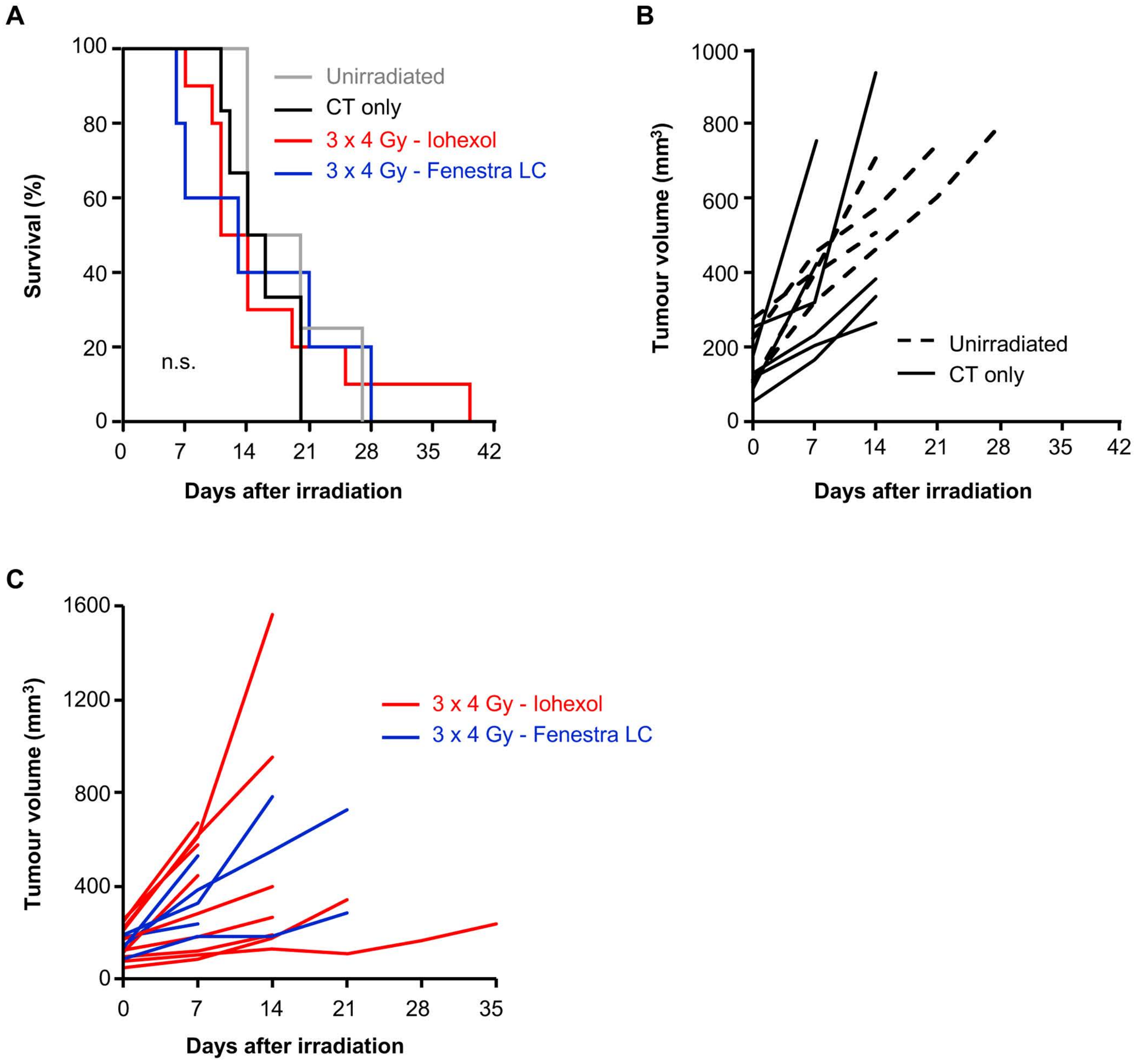

Figure S2

Disease Models & Mechanisms • Supplementary information

Fig. S2. Contrast agent does not affect response to radiotherapy in tumour-bearing KPC mice.

A) Kaplan-Meier survival analysis of tumour-bearing KPC mice left unirradiated (grey line, n = 4 mice), exposed to 18.66 cGy during CT scan acquisition on day 0, 2 and 4 (black line, n = 6 mice) or SARRP-irradiated with three fractions of 4 Gy on days 0, 2 and 4 using either iohexol (red line, n = 10 mice) or Fenestra LC (blue line, n = 5 mice) as CT contrast agents. Statistical significance was tested in pairwise comparisons using the Log-Rank test. n.s. = non-significant $p > 0.05$.

B) Tumour volume monitoring by weekly 3D ultrasound scans of tumour-bearing KPC mice left unirradiated (dashed line, n = 4 mice) or exposed to 18.66 cGy during CT scan acquisition on day 0, 2 and 4 (solid lines, n = 6 mice). Each line represents an individual mouse. Tumour volume data shown in Figure 5B are included here.

C) Tumour volume monitoring by weekly 3D ultrasound scans of tumour-bearing KPC mice that received 3 x 4 Gy irradiation on day 0, 2 and 4, targeted to the tumour using iohexol (red lines, n = 10 mice) or Fenestra LC (blue lines, n = 5 mice) for CT contrast. Each line represents an individual mouse. Tumour volume data shown in Figure 5B are included here.

Disease Models & Mechanisms • Supplementary information

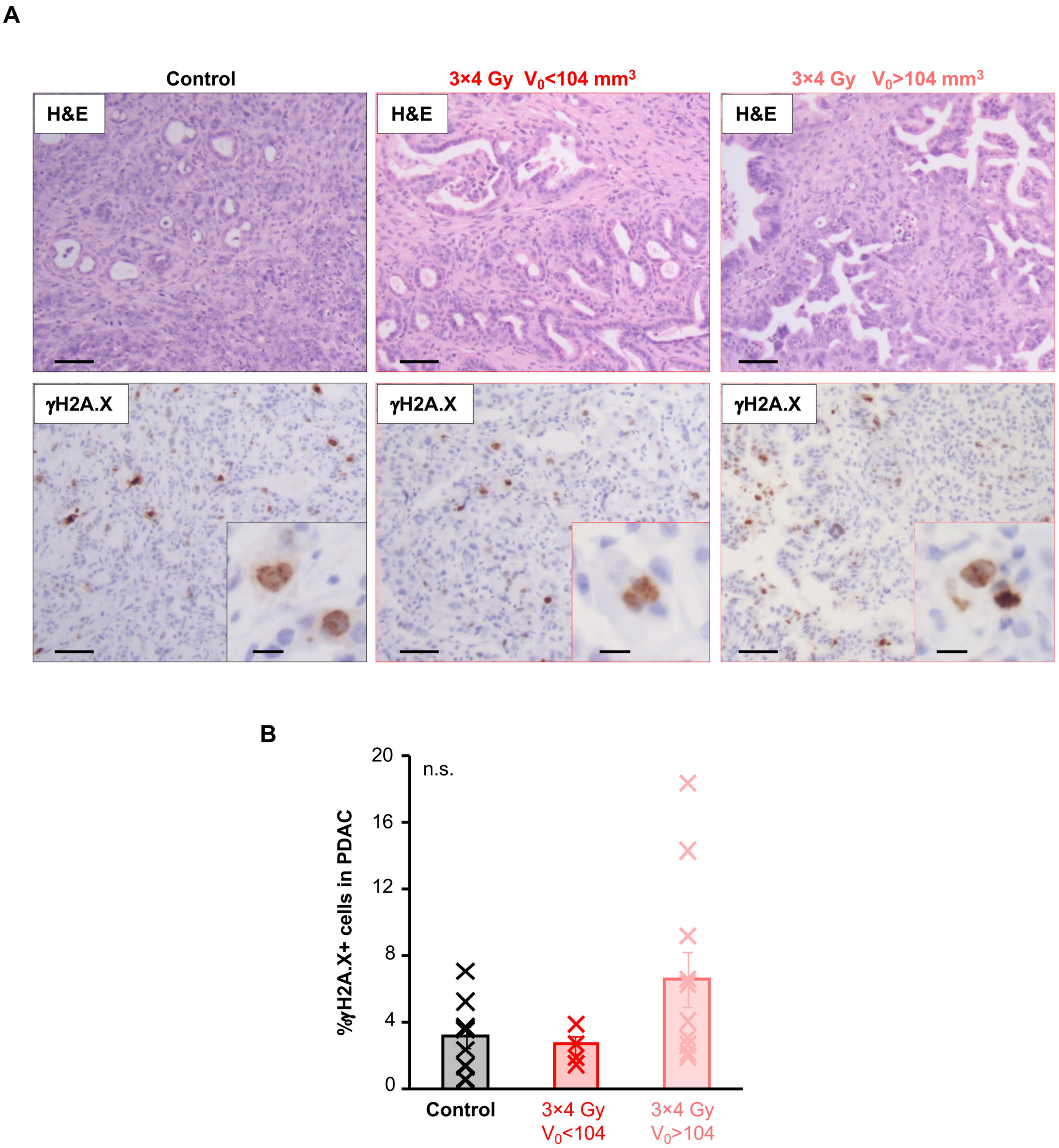

Figure S3

Disease Models & Mechanisms • Supplementary information

Fig. S3. Tumour size at the time of irradiation does not affect the level of DNA damage in irradiated tumours.

A) Representative images of immunohistochemical staining for γH2A.X on sections of PDAC harvested at endpoint from control KPC mice (n = 10) or KPC mice irradiated with 3 x 4 Gy with an initial tumour volume of < 104 mm^3 (n = 4) or with 3 x 4 Gy with an initial tumour volume of > 104 mm^3 (n = 11), which were stained with haematoxylin and eosin (H&E, top panels) or for the marker of DNA double-strand break γH2A.X (bottom panels). Main image scale bars = 50 μm, inset image scale bars = 10 μm.

B) Quantification of the proportion of γH2A.X-positive (γH2A.X+) PDAC cells using HALO software in control KPC mice (n = 10) or KPC mice irradiated with 3 x 4 Gy with an initial tumour volume of < 104 mm^3 (n = 4) or with 3 x 4 Gy with an initial tumour volume of > 104 mm^3 (n = 11). Each cross represents an individual mouse. Bar represents mean γH2A.X score +/- SEM. Statistical significance tested using one-way ANOVA with Bonferroni correction. n.s. = non-significant $p > 0.05$.

Disease Models & Mechanisms • Supplementary information

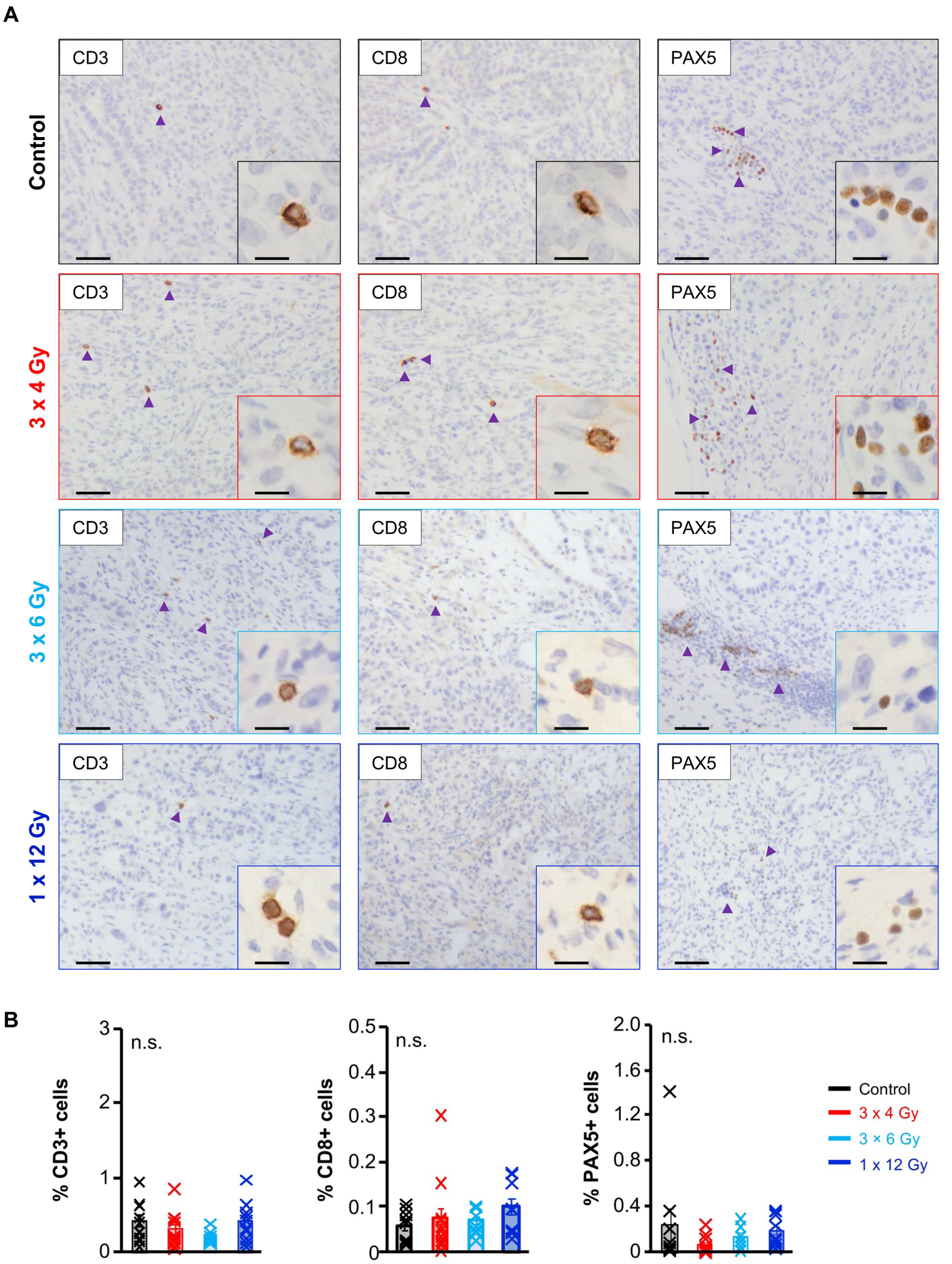

Figure S4

Disease Models & Mechanisms • Supplementary information

Fig. S4. Immune cell content in tumours from unirradiated or irradiated tumour-bearing KPC mice.

A) Representative images of immunohistochemical staining for CD3 (T cells), CD8 (CTLs) and PAX5 (B cells) in PDAC harvested at endpoint from control KPC mice (n = 10) or from KPC mice irradiated with 3 fractions of 4 Gy (red, n = 12), three fractions of 6 Gy (cyan, n = 7) or 1 fraction of 12 Gy (blue, n = 10). Immunopositivity is brown and nuclei are counterstained in blue. Arrowheads indicate the location of isolated T cells (CD3), CTLs (CD8) or B cells (PAX5). Main image scale bars = 50 μm, inset image scale bars = 10 μm.

B) HALO software was used to quantify the proportion of T cells (CD3+), CTLs (CD8+) and B cells (PAX5+) in stained PDAC sections as described in panel A. Each cross represents an individual mouse. The bars represent the mean score +/- SEM. Statistical significance was tested using one-way ANOVA with Bonferroni correction. n.s. = non-significant $p > 0.05$.

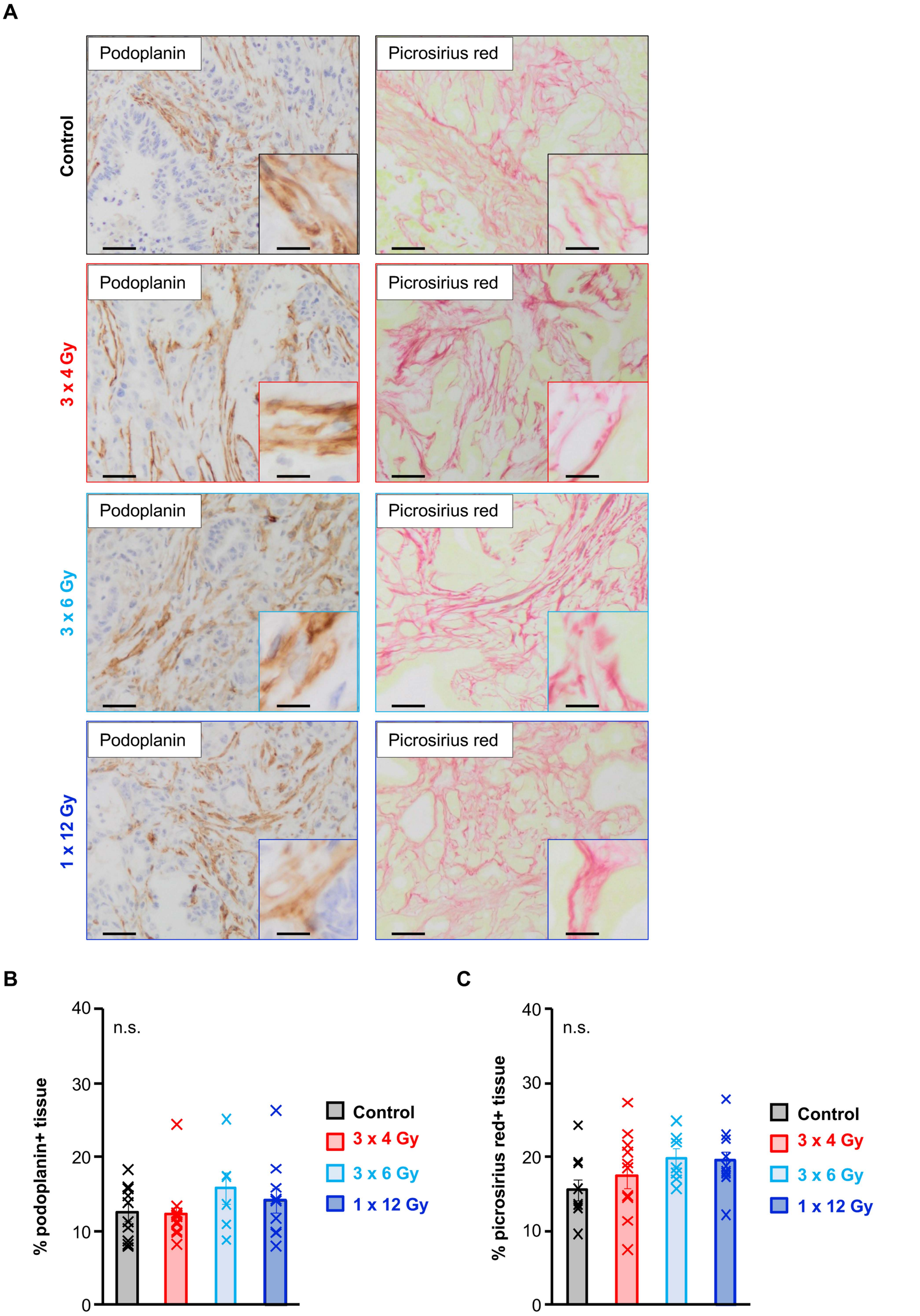

Figure S5

Disease Models & Mechanisms • Supplementary information

Fig. S5. The stromal and fibrotic content of SARRP-irradiated PDAC in KPC mice.

A) Representative images of immunohistochemical staining for the pan-fibroblast marker podoplanin (PDPN) and of staining for collagen with picrosirius red in PDAC harvested at endpoint from control KPC mice (n = 10) or from KPC mice irradiated with three fractions of 4 Gy on day 0, 2 and 4 (red, n = 12), three fractions of 6 Gy on day 0, 2 and 4 (cyan, n = 7) or 1 fraction of 12 Gy on day 0 (blue, n = 10). Main image scale bars = 50 μm, inset image scale bars = 10 μm.

B) HALO software was used to quantify the proportion of podoplanin-positive (podoplanin+) cells within PDAC of the mice from panel A. Each cross represents an individual mouse. Bar represents mean score +/- SEM. Statistical significance tested using one-way ANOVA with Bonferroni correction. n.s. = non-significant $p > 0.05$.

C) HALO software was used to quantify the proportion of picrosirius red-positive (picrosirius red+) tissue within PDAC of the mice from panel A. Each cross represents an individual mouse. Bar represents mean score +/- SEM. Statistical significance tested using one-way ANOVA with Bonferroni correction. n.s. = non-significant $p > 0.05$.

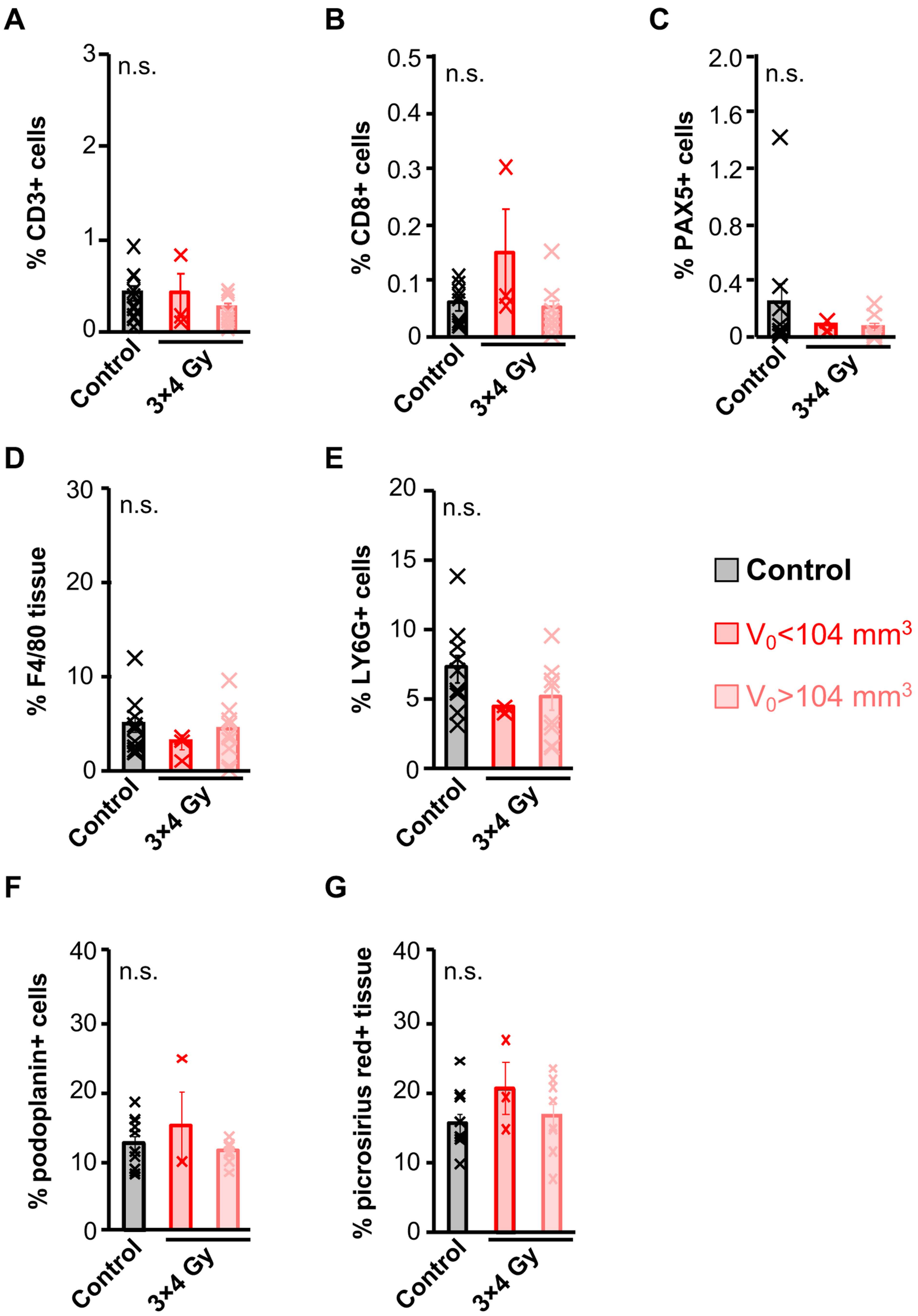
A
% CD3+ cells
n.s.
3
2
1
0
Control
3x4 Gy
B
% CD8+ cells
n.s.
0.5
0.4
0.3
0.2
0.1
0
Control
3x4 Gy
C
% PAX5+ cells
n.s.
2.0
1.6
1.2
0.8
0.4
0
Control
3x4 Gy
D
% F4/80 tissue
n.s.
30
20
10
0
Control
3x4 Gy
E
% LY6G+ cells
n.s.
20
15
10
5
0
Control
3x4 Gy
Control
V0<104 mm3
V0>104 mm3
F
% podoplanin+ cells
n.s.
40
30
20
10
0
Control
3x4 Gy
G
% picrosirius red+ tissue
n.s.
40
30
20
10
0
Control
3x4 Gy

Figure S6

Fig. S6. The effect of tumour volume at the time of irradiation on the composition of the immune microenvironment of KPC tumours.

Quantification, using HALO software, of the proportion of (A) T cells (CD3+), (B) cytotoxic T cells (CD8+), (C) B cells (PAX5+), (D) macrophages (F4/80+), (E) neutrophils (LY6G+), (F) fibroblasts (podoplanin+) and (G) collagen (picrosirius red+) in PDAC harvested at endpoint from control KPC mice (n = 10), KPC mice with an initial tumour volume < 104 mm^3 irradiated with 3 x 4 Gy (n = 3), or KPC mice with an initial tumour volume > 104 mm^3 irradiated with 3 x 4 Gy (n = 9). Each cross represents an individual mouse. Bar represents mean immune score +/- SEM. Statistical significance tested using one-way ANOVA with Bonferroni correction. n.s. = non-significant p > 0.05.

Table S1. Properties of the iodine contrast agents Iohexol and Fenestra LC.

Contrast agent	Stock iodine concentration	Volume administered	Route of administration	Additional
Iohexol	350 mg/ml	250 µl (150 µl IV), undiluted (88mg I)	IP, PO, IV	n/a
Fenestra LC	50 mg/ml	10 µl per gram body weight, undiluted (0.5mg I/g)	IV	Subcutaneous 200 µl PBS injection for hydration prior to Fenestra LC administration.

Table S2. List of antibodies used for immunohistochemistry.

Antibody target	Clone	Antibody dilution	Supplier
Podoplanin	ab11936	1/4000	Abcam
CD8	4SM 95	1/500	eBioscience
CD3	SP7	1/100	Abcam
Ly6G	IA8	1/60000	BioXcell
γH2A.X	20 E3	1/120	Cell Signalling Technology
PAX5	ab109443	1/1000	Abcam
F4/80	CI:A3-1	1/100	Abcam
